# LINC00922 promotes deterioration of gastric cancer

**DOI:** 10.1371/journal.pone.0267798

**Published:** 2022-05-05

**Authors:** Hua Ge, Yan Yan, Fei Tian, Lingfei Guo, Xueyan He, Can Wang, Jiacheng Song, Zhilong Deng

**Affiliations:** 1 Department of Gastrointestinal Surgery, The Third Affiliated Hospital of Zunyi Medical University (The First People’s Hospital of Zunyi), Zunyi, Guizhou, People’s Republic of China; 2 Quality Control Department, The Third Affiliated Hospital of Zunyi Medical University (The First People’s Hospital of Zunyi), Zunyi, Guizhou, People’s Republic of China; Children’s Cancer Institute Australia, AUSTRALIA

## Abstract

Several studies have demonstrated the association of lncRNAs with a variety of cancers. Here, we explored the role of LINC00922 in gastric cancer (GC) using bioinformatics approaches and in vitro experiments. We examined the expression of LINC00922 and the prognosis of GC patients based on data from The Cancer Genome Atlas (TCGA) and Gene Expression Profiling Interactive Analysis (GEPIA). LINC00922-related genes were identified by the Multi Experiment Matrix (MEM) database and The Atlas of Noncoding RNAs in Cancer (TANRIC), followed by Gene Ontology (GO), Kyoto Encyclopedia of Genes and Genomes (KEGG) and protein-protein interaction analysis. The significance of LINC00922 in cell proliferation, apoptosis, invasion and migration was assessed by MTT assay, flow cytometry, Transwell assay and wound-healing assay. The expression of LINC00922 was increased in GC tissues compared with adjacent non-tumor tissues, and increased LINC00922 expression was correlated with poor overall survival and disease-free survival. In addition, 336 overlapping genes were identified by the MEM database and TANRIC and found to be involved in GC-related biological processes, such as cell adhesion and migration, as well as TGF-β signaling. In the protein-protein interaction network, hub genes, such as FSTL3 and LAMC1, were identified. LINC00922 overexpression significantly promoted cell proliferation and invasion in vitro, whereas LINC00922 knockdown exerted opposite effects. In summary, our findings indicate that LINC00922 is overexpressed in GC tissues, suggesting that it might play a role in the development and progression of GC, and thus, it might serve as a prognostic indicator of GC.

## Introduction

Gastric cancer is the fifth most common cancer and the third most fatal cancer worldwide. In 2018, there were more than one million cases of GC, which caused an estimated 783,000 deaths. As such, GC is a public health-related burden for developed and developing countries [1]. Almost half of the world’s GC cases and deaths occur in China, with an estimated 500,000 deaths in 2015 [2]. GC is a multi-factorial disease, and both environmental (68%) and genetic (22%) factors have been reported to associate with its etiology [3]. Personal lifestyle choices, such as insufficient fruits and vegetables in the diet, excessive alcohol consumption and high intake of salt, have been demonstrated to associate with GC [4–6]. In addition, a family history of GC and *Helicobacter pylori* infection both increase the risk of developing GC [7,8]. As most patients have non-specific symptoms in the early stages of the disease, GC can progress to advanced stages, which is when most patients are diagnosed. Therefore, it is important to identify reliable diagnostic and prognostic markers of GC, with the hopes of detecting GC in the early stages of the disease.

Long non-coding RNAs (lncRNAs) are a group of non-coding RNAs of more than 200 nucleotides in length without open reading frames [9]. LncRNAs have a variety of functions, including post-transcriptional regulation, gene regulation and intercellular signaling [10,11], and dysregulation of lncRNAs is involved in a variety of diseases such as cancer [12]. Accumulating evidence has revealed that lncRNAs are essential for cancer progression and metastasis [13,14]. Recently, new lncRNAs have been identified, and bioinformatics approaches have been used to understand the roles of lncRNAs [15,16]. However, the function of LINC00922 in the development of GC is unclear. Thus, we investigated the role of LINC00922 in GC using data mining and in vitro experiments.

## Results

### Expression and prognostic value of LINC00922 in GC based on TCGA data

**Fig 1:**
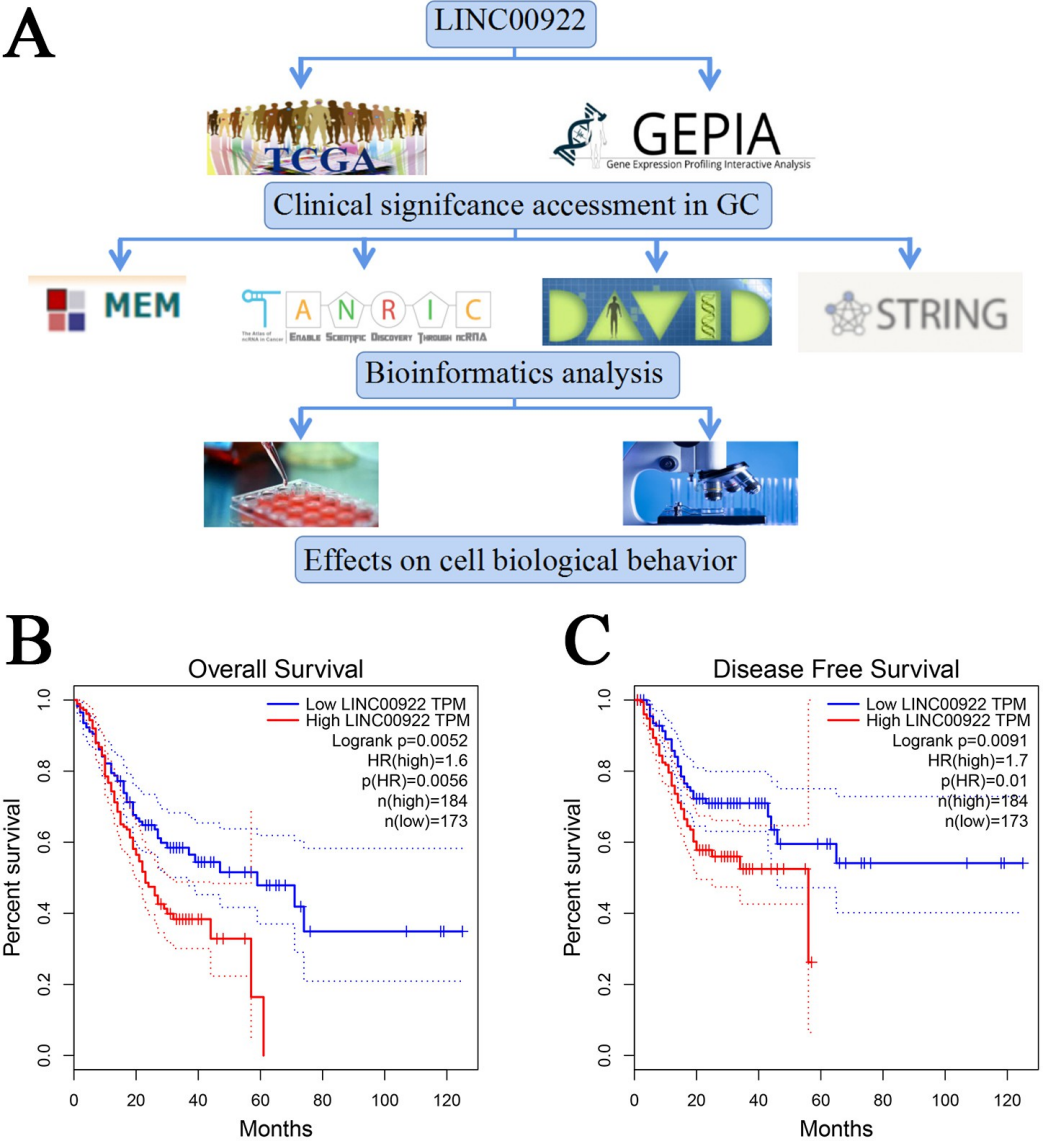
Flow chart and Kaplan-Meier curves for survival of LINC00922 from GEPIA. (A) Flow chart. (B) Overall survival. (C) Disease-free survival.

A flow chart of the study design is shown in Fig 1A. In total, we extracted 407 samples from TCGA datasets, including 375 tumor and 32 non-tumor samples. As shown in Table 1, LINC00922 expression was increased in GC tissues (2.908 ± 0.077) and decreased in adjacent non-tumor tissues (0.790 ± 0.189) (P < 0.001). However, LINC00922 expression was not correlated with age, gender, race, tumor depth, lymphnode invasion, remote metastasis, and pathological stage. Next, we used GEPIA to determine the prognostic value of LINC00922 in GC. As shown in Fig 1B and 1C, increased LINC00922 expression was significantly correlated with poor overall survival (P = 0.0056) and disease-free survival (P = 0.0091), indicating that LINC00922 functions as an oncogene in GC.

**Table 1:** Expression of LINC00922 and clinicopathological features in GC patients in TCGA.

Clinicopathological features	n	LINC00922 expression	t	P
		Mean ± SD		
Tissue			7.846	< 0.001
GC	375	2.908 ± 0.077		
Adjacent non-tumour	32	0.790 ± 0.189		
Age			0.573	0.567
<60	112	2.976 ± 0.150		
≥60	263	2.880 ± 0.090		
Gender			0.714	0.476
Male	241	2.867 ± 0.099		
Female	134	2.982 ± 0.121		
Race			F = 1.441*	0.238
Black	12	2.311 ± 0.293		
White	255	2.880 ± 0.090		
Asian	108	3.042 ± 0.158		
AJCC pathologic T			F = 2.306*	0.101
T1-T2	99	2.683 ± 0.163		
T3-T4	268	2.970 ± 0.089		
TX	8	3.632 ± 0.305		
AJCC pathologic N			F = 0.283*	0.754
N0	112	2.870 ± 0.143		
N1-N2	246	2.908 ± 0.096		
NX	17	3.163 ± 0.312		
AJCC pathologic M			F = 1.391*	0.250
M0	330	2.862 ± 0.083		
M1	25	3.201 ± 0.313		
MX	20	3.316 ± 0.233		
Pathologic stage			0.860	0.390
I-II	169	2.835 ± 0.112		
III-IV	206	2.969 ± 0.107		

Abbreviations: GC, Gastric cancer. TCGA, The Cancer Genome Atlas. SD, Standard deviation.

*, one-way analysis was performed.

### Prediction of genes related to LINC00922

**Fig 2:**
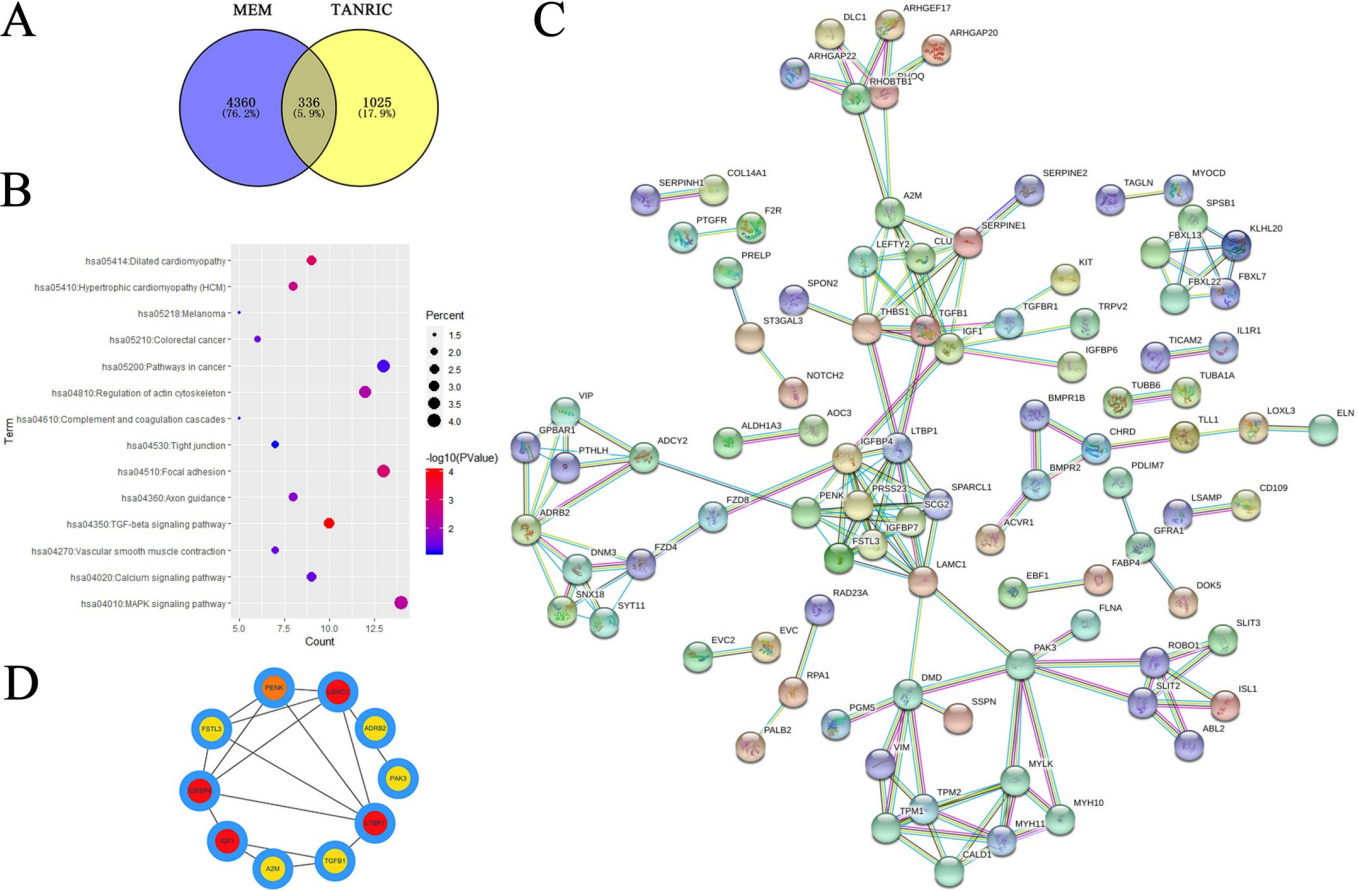
Enrichment analysis of genes related to LINC00922 in gastric cancer. (A) Venn diagram of the overlapping genes predicted by the MEM database and TANRIC. (B) KEGG pathway enrichment analysis by DAVID. (C) Protein-protein interaction network of genes related to LINC00922. (D) Top ten hub genes of LINC00922.

To identify the genes related to LINC00922, the MEM database and TANRIC were used. For the MEM database, 4,360 related genes were identified when the threshold was set to 10^−6^. For TANRIC, 1,025 related genes were identified. Among these, 336 genes were overlapping (Fig 2A).

### GO and KEGG analyses

We used Database for Annotation, Visualization and Integrated Discovery (DAVID) to conduct GO and KEGG analyses. For the GO database, there are three categories of functional annotations, namely, molecular function (MF), cellular component (CC) and biological process (BP). As shown in Table 2, there were 10, 10 and 3 significant terms in BP, CC and MF, respectively (false discovery rate (FDR) < 0.05). For MF, cell adhesion, biological adhesion and cell motion were mostly enriched. For CC, plasma membrane, actin cytoskeleton and contractile fiber part were mostly enriched. For BP, actin binding, cytoskeletal protein binding and growth factor binding were mostly enriched. In the KEGG pathways, there were 11 significant terms (P < 0.05) (Table 3 and Fig 2B). Among these, the TGF-β signaling pathway, dilated cardiomyopathy and focal adhesion were the top three terms.

**Table 2:** The GO analysis of predicted target genes of LINC00922.

GO ID	Term	Count	FDR
Biological process			
0007155	Cell adhesion	36	3.36E-04
0022610	Biological adhesion	36	3.40E-04
0006928	Cell motion	28	8.04E-04
0030036	Actin cytoskeleton organization	17	1.31E-02
0007010	Cytoskeleton organization	24	2.03E-02
0048812	Neuron projection morphogenesis	16	2.70E-02
0030029	Actin filament-based process	17	2.94E-02
0040012	Regulation of locomotion	15	3.52E-02
0016477	Cell migration	18	4.24E-02
0051094	Positive regulation of developmental process	18	4.64E-02
Cellular component			
0005886	Plasma membrane	115	5.95E-05
0015629	Actin cytoskeleton	22	9.38E-05
0044449	Contractile fiber part	13	2.75E-03
0005576	Extracellular region	69	2.76E-03
0044421	Extracellular region part	41	5.23E-03
0043292	Contractile fiber	13	5.66E-03
0030016	Myofibril	12	1.44E-02
0005615	Extracellular space	32	1.46E-02
0030017	Sarcomere	11	2.81E-02
0030018	Z disc	8	3.73E-02
Molecular function			
0003779	Actin binding	24	1.06E-04
0008092	Cytoskeletal protein binding	30	1.74E-04
0019838	Growth factor binding	12	7.48E-03

In the GO analysis of predicted target genes of LINC00922 in 2 databases, there were 10 available biological processes, 10 cellular components, 3 molecular functions (FDR < 0.05). GO, Gene Ontology. FDR, false discovery rate.

**Table 3:** Pathway analysis of the predicted target genes of LINC00922.

Title	Count	P	Genes
TGF-β signaling pathway	10	9.36E-05	LTBP1, CDKN2B, TGFBR1, LEFTY2, BMPR2, BMPR1B, THBS1, CHRD, TGFB1, ACVR1
Dilated cardiomyopathy	9	7.65E-04	ITGA9, ADCY2, ITGA5, DMD, IGF1, TPM2, CACNA1C, TPM1, TGFB1
Focal adhesion	13	1.21E-03	CAV2, IGF1, FLNC, FLNA, ITGA9, ITGA5, PAK3, PDGFRA, TNN, LAMC1, THBS1, MYLK, AKT3
Hypertrophic cardiomyopathy (HCM)	8	2.25E-03	ITGA9, ITGA5, DMD, IGF1, TPM2, CACNA1C, TPM1, TGFB1
MAPK signaling pathway	14	4.69E-03	IL1R1, FGF7, TGFBR1, MRAS, FGF13, FLNC, FLNA, TGFB1, BDNF, PDGFRA, CACNA1H, NFATC4, CACNA1C, AKT3
Regulation of actin cytoskeleton	12	6.40E-03	ITGA9, FGF7, PAK3, ITGA5, CFL2, MRAS, PDGFRA, RDX, FGF13, MYLK, MYH10, F2R
Axon guidance	8	2.11E-02	PAK3, ROBO1, CFL2, SEMA3C, NFATC4, UNC5C, SLIT2, SLIT3
Vascular smooth muscle contraction	7	3.36E-02	ADCY2, CALD1, MYH11, NPR2, CACNA1C, KCNMB1, MYLK
Colorectal cancer	6	3.46E-02	FZD8, TGFBR1, PDGFRA, FZD4, AKT3, TGFB1
Calcium signaling pathway	9	3.59E-02	ADRB2, ADCY2, PDE1A, PDGFRA, CACNA1H, PTGFR, CACNA1C, MYLK, F2R
Pathways in cancer	13	4.97E-02	FZD8, FGF7, CDKN2B, TGFBR1, PDGFRA, IGF1, FGF13, KIT, LAMC1, HHIP, FZD4, TGFB1, AKT3

The pathway analysis was performed in Kyoto Encyclopedia of Genes and Genomes database and there were 11 available pathways were significant (P < 0.05).

### Construction and analysis of protein-protein interaction network

**Fig 3:**
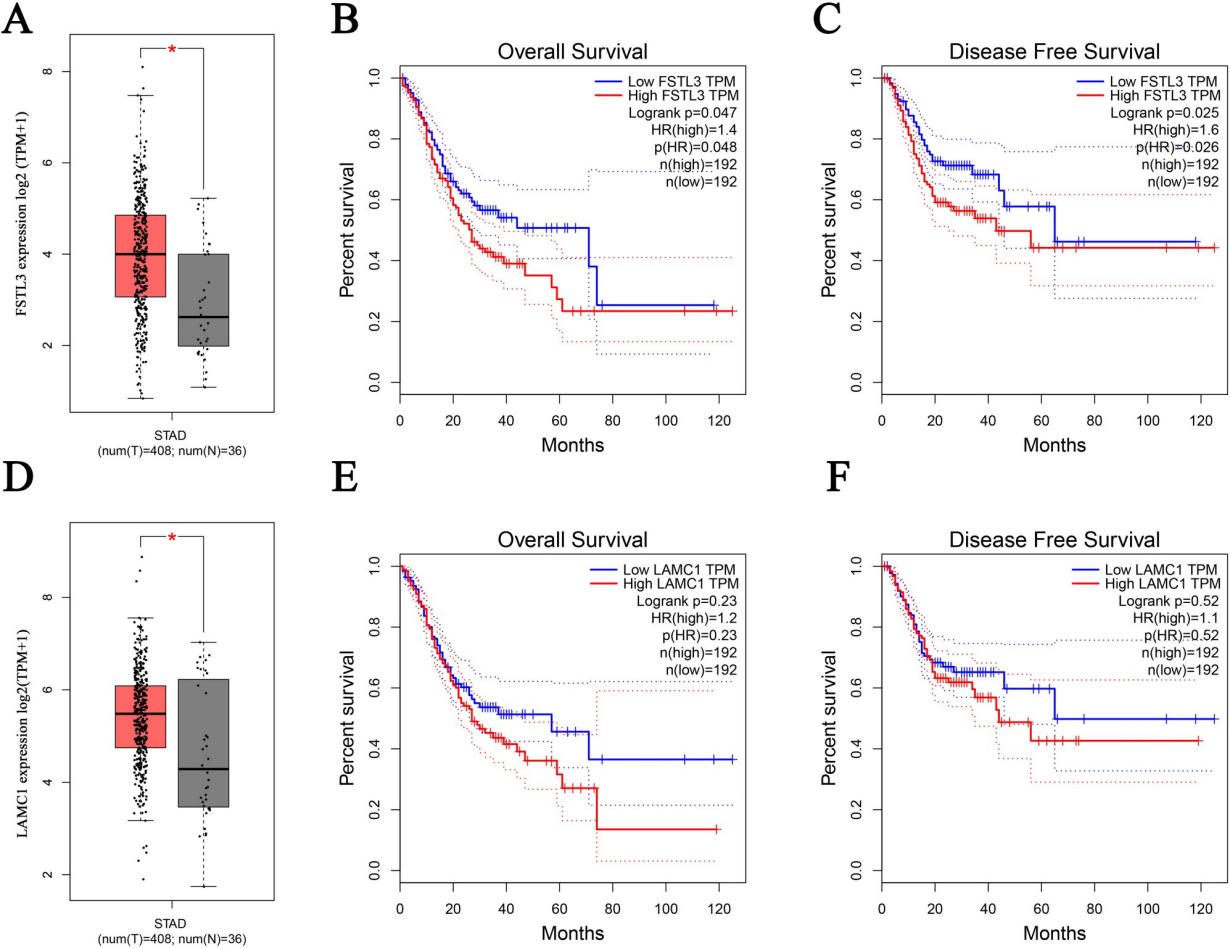
Expression level, overall survival and disease-free survival of FSTL3 and LAMC1 in GC patients from GEPIA. (A) Expression level of FSTL3. (B) Overall survival of FSTL3. (C) Disease-free survival of FSTL3. (D) Expression level of LAMC1. (E) Overall survival of LAMC1. (F) Disease-free survival of LAMC1.

As shown in Fig 2C, the protein-protein interaction network contained 335 nodes and 167 edges, and the degree of connectivity was analyzed by Cytoscape using these features. The genes with more than three degrees were defined as hub genes. As shown in Fig 2D, LAMC1, LTBP1, IGFBP4, IGF1, PENK, SPARCL1, PRSS23, FSTL3, PAK3 and ADRB2 were the top ten hub genes. Next, we examined the expression levels and prognostic values of the most significant hub genes in GEPIA. The results indicated FSTL3 was highly expressed in GC tissues, and correlated with overall survival and disease-free survival (Fig 3A–3C), while LAMC1 expression was increased in GC tissues (Fig 3D), but not correlated with overall survival and disease-free survival of GC patients (Fig 3E and 3F). Thus, FSTL3 and LAMC1 are the likely targets of LINC00922.

### LINC00922 is essential for GC cell proliferation

**Fig 4:**
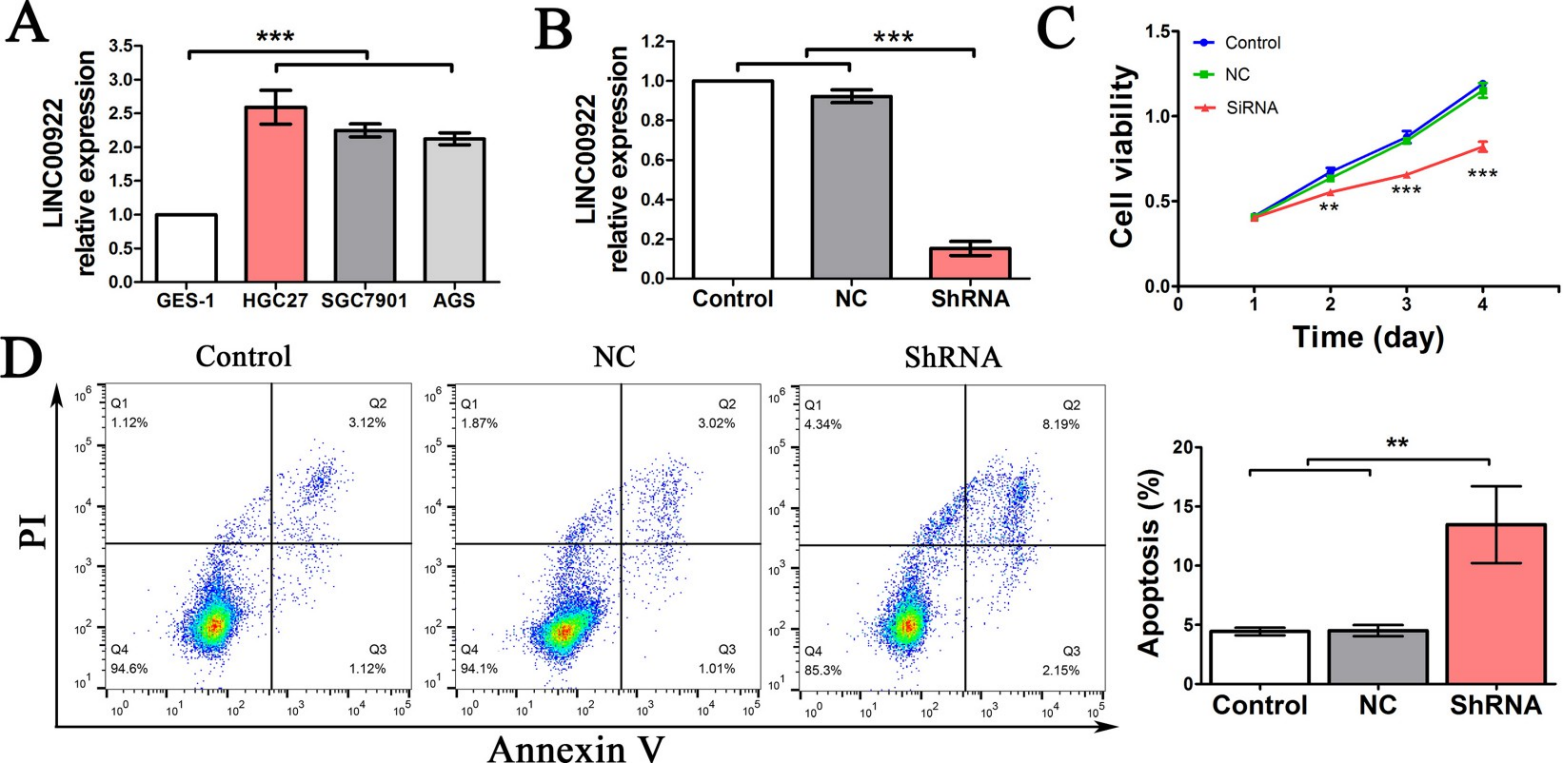
Expression of LINC00922 and GC cell proliferation and apoptosis. (A) Expression of LINC00922 in different GC cell lines, as assessed by RT-PCR. (B) LINC00922 expression in HGC27 cells was examined by RT-PCR. LINC00922 expression was decreased in the ShRNA group compared with the control group (P < 0.05). (C) Cell growth curve of control cells and those transfected with shRNAs and NC. (D) Cell apoptosis was determined by flow cytometry. The apoptosis rate was increased in the ShRNA group compared with the control group (P < 0.05). **P < 0.01, and ***P < 0.001.

To examine the function of LINC00922, we assessed the expression of LINC00922 in GC cell lines HGC27, SGC7901 and AGS as well as the normal gastric cell line GES-1. As shown in Fig 4A, all three GC cell lines expressed a high level of LINC00922, and the HGC27 cell line expressed the highest level of expression. Next, we silenced LINC00922 in HGC27 cells (Fig 4B). To assess cell proliferation, the MTT assay was used, and the results showed that LINC00922 knockdown could significantly affect cell viability in the ShRNA group (P < 0.05) (Fig 4C). Furthermore, decreased LINC00922 expression could increase HCG27 cell apoptosis (P < 0.05) (Fig 4D). These findings indicate that LINC00922 is important in cell proliferation.

### LINC00922 associates with gastric cancer cell invasion and migration

**Fig 5:**
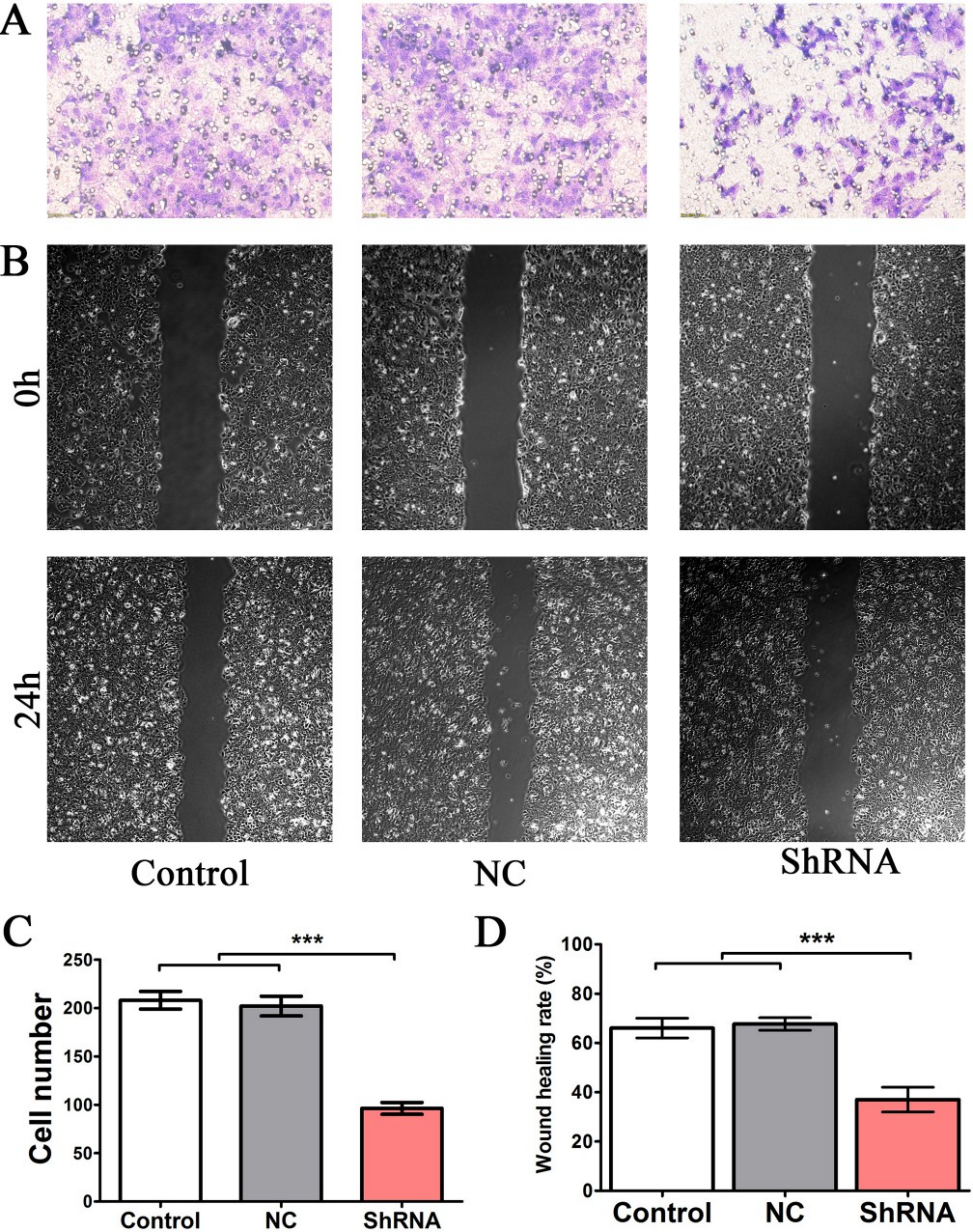
Effects of LINC00922 knockdown on HCG27 cell invasion. (A) Representative images of the Transwell assay of control cells and those transfected with shRNAs and NC. (B) Representative images of the wound-healing assay showing that cell migration was inhibited in cells transfected with shRNAs. (C) Quantification of Transwell assays in control cells and those transfected with shRNAs and NC. (D) Quantification of wound-healing assays in control cells and those transfected with shRNAs and NC. ***P < 0.001.

The Transwell assay was performed to assess cell invasion. As shown in Fig 5A and 5C, decreased LINC00922 expression could inhibit cell invasion. Next, the wound-healing assay was used to evaluate the effects of LINC00922 on cell migration. As shown in Fig 5B and 5D, LINC00922 knockdown could significantly decrease cell migration. These results reveal that LINC00922 is an important regulator of cell invasion and migration.

## Discussion

According to The National Central Cancer Registry of China, the mortality rate of patients with GC is the second highest among all cancers in China [17]. Although advances have been made in the treatment of GC, such as surgery, chemotherapy and radiotherapy, the prognosis of GC patients still remains poor. Among the prognostic and therapeutic biomarkers, HER2 is the only biomarker screened in clinical settings. However, HER2 is overexpressed in only 9%–38% of GC patients [18]. Thus, a better understanding of GC will lead to the identification of new biomarkers.

Our results indicated that LINC00922 was overexpressed in GC tissues. Recently, several studies have demonstrated the association of lncRNAs with a variety of cancers. For example, the increased expression of lncRNA HOXA11-AS has been reported to associate with cell cycle progression, invasion and migration in GC [19]. Another lncRNA MALAT1, regulates VE-cadherin/β-catenin, ERK/MMP and FAK/paxillin pathways to facilitate angiogenesis and promote metastasis [20]. Here, we demonstrated that LINC00922 was overexpressed in GC tissues and correlated with poor prognosis in GC patients. These findings are consistent with those of other studies, which reported several lncRNAs, such as PEG10, MT1JP and BLACAT1, to associate with cancer patient survival [21–23].

We identified 366 genes related to LINC00922 by the MEM database and TANRIC. By GO and KEGG analyses, adhesion-related processes, such as cell and biological adhesion, were significantly enriched. Cell migration was also related to LINC00922, which was confirmed by in vitro experiments that showed LINC00922 to be required for cell invasion and migration. To date, several studies have revealed that lncRNAs play critical roles in metastasis. For example, Sun et al. demonstrated that YAP1 is highly up-regulated in GC, accelerates tumor growth and metastasis through ERK1/2 phosphorylation and regulates lncRNAs, including HOTAIR, MALAT1, LATS2 and LATS2-AS1-001 [24]. HOTAIR and LINC00261 can regulate the epithelial-mesenchymal transition by modulating the expression of E-cadherin, N-cadherin and vimentin [25,26].

It is well established that an imbalance between cell proliferation and survival is critical for cancer progression. For example, H19 is up-regulated in GC and suppresses the expression of p53 and p53 target B-cell lymphoma-associated X protein, which induce GC cell proliferation [27]. H19 also regulates GC cell proliferation by acting as a competing endogenous RNA of miR-141 and competing for binding to its target genes, including insulin-like growth factor (IGF) 1, IGF receptor 2 and zinc finger E-box-binding homeobox 1 [28]. Gastric carcinoma high expressed transcript (GHET1) interacts with IGF2 mRNA binding protein 1 (IGF2BP1), which enhances the interaction between IGF2BP1 and c-Myc and increases the expression of c-Myc, thereby promoting cell proliferation [29]. Our findings demonstrate that LINC00922 inhibits cell apoptosis and promotes cell proliferation, indicating that the expression of LINC00922 might disrupt several cancer-related processes and signaling pathways, and contribute to GC.

We investigated the prognostic significance of LINC00922 in GC using GEPIA, and the results revealed that increased LINC00922 expression correlated with shorter overall survival and disease-free survival. In addition, we obtained the predicted hub genes, FSTL3 and LAMC1, of LINC00922 using STRING and Cytoscape. FSTL3 is a member of the follistatin family, which binds to and inactivates activin to regulate cell growth and differentiation [30]. FSTL3 has been reported to participate in tumorigenesis and associate with nuclear grade and tumor size in breast cancer. LAMC1 encodes laminin γ1, which is involved in a variety of processes such as normal tissue development, cancer cell invasion and metastasis [31,32]. In hepatocellular carcinoma (HCC), LAMC1 competes for miR-124 binding and acts as a trans-regulator to induce the expression of CD151 [33]. Zhang et al. reported that overexpression of LAMC1 in HCC enhances invasion and migration and predicts poor prognosis [34]. Taken collectively, these findings suggest that FSTL3 and LAMC1 are involved in tumor biology and targeting LINC00922 may be an effective therapy in GC patients.

There were several limitations in this study. First, lncRNAs play a variety of roles in GC, many of which were not examined here. As such, the functions of LINC00922, such as the ceRNA mechanism, should be further explored and combined with bioinformatics approaches and in vitro and in vivo experiments [35–39]. Second, a larger cohort and a computational model are needed to assess the potential of LINC00922 as a biomarker for GC [40–42]. Despite these limitations, our results indicate that LINC00922 is critical in the progression, invasion and migration of GC cells and can be used as a potential target for the treatment of GC.

## Materials and methods

### Data collection

GC tissue and non-tumor tissue gene expression profiles were obtained from TCGA dataset (https://portal.gdc.cancer.gov/). To normalize the expression levels of lncRNAs in different samples, the data were log2-scaled. Differential expression analysis of lncRNAs was performed with the Limma package (http://www.bioconductor.org/). P-value < 0.05 and fold change (FC) > 1 were set as the threshold. Among the differentially expressed lncRNAs, LINC00922 was selected for further study.

### GEPIA dataset analysis

GEPIA is an online web server that includes 9,736 tumor tissue and 8,587 normal samples tissue specimens in TCGA and genotype-tissue expression datasets. Differential gene expression analysis and Kaplan-Meier analysis of overall survival and disease free survival were conducted by GEPIA.

### Genes potentially related to LINC00922 in gastric cancer

Online bioinformatics databases MEM and TANRIC were used to predict the genes potentially related to LINC00922 [43,44]. In this study, overlapping genes from the aforementioned programs were recorded as target genes of LINC00922 in GC.

### Analysis of associated gene-enriched pathways and their functions

DAVID (https://david.ncifcrf.gov/) was used to conduct GO and KEGG analyses [45]. The results were considered to be significant when the FDR was < 0.05 in GO analysis and the P-value was < 0.05 in KEGG analysis.

### Protein-protein interaction network construction and confirmation of hub genes

To identify the interactions between overlapping genes, STRING 11.0 (http://www.string-db.org) was used to construct the protein-protein interaction network [46]. Genes with combined scores > 0.9 were obtained. The degree of connectivity in the protein-protein interaction network was analyzed with Cytoscape software 3.0, and the hub gene network of LINC00922 was constructed. In the protein-protein interaction network, each node indicated a protein, and each color corresponded to a cluster. The edges represented the functional associations of prediction, and the thickness of lines represented the strength of evidence.

### Cell culture and transfection

HGC27, SGC7901 and AGC cell lines were obtained from the Cancer Hospital of the Chinese Academy of Medical Sciences (China). The GES-1 cell line was obtained from the American Type Culture Collection (USA). The cells were cultured in Roswell Park Memorial Institute (RPMI) medium supplemented with 10% fetal bovine serum (FBS) (Gibco, USA) and 100 μg/ml streptomycin (Invitrogen, USA), and the cells were cultured in conditions of 5% CO_2_ at 37˚C.

The LINC00922 sequence was obtained from GenBank, and shRNAs against LINC00922 were synthesized with the following sequences: shRNA-1 (5’-CCTGCACCTACAGATCTACACCTCGAGGTGTAGATCTGTAGGTGCAGG-3’), shRNA-2 (5’-TGCAGGAAGTGTTCATCTAAGCTCGAGCTTAGATGAACACTTCCTGCA-3’). ShRNAs against LINC00922 or the negative control were transfected into the pSICOR-GFP plasmid (Shanghai Geneline Bioscience, China). Flow cytometry was used to confirm knockdown. The cells with the highest knockdown efficiency were used in subsequent experiments.

### Real-time polymerase chain reaction (RT-PCR)

Total RNA was isolated from cells using TRIzol reagent (Invitrogen, USA) according to the manufacturer’s instructions, and cDNA was synthesized. Next, the mRNA transcripts were amplified in the Eppendorf PCR system (China). The relative expressions of each target gene was normalized to that of GADPH. The primer sequences were as follows: LINC00922 (5’-CAAGACCGCCAAGCATTAAGAT-3’ [forward] and 5’-AGTGGCTCCTCCACCACCTCCT-3’ [reverse]) and GADPH (5’-GTCAACGGATTTGGTCTGTATT-3’ [forward] and, 5’-AGTCTTCTGGGTGGCAGTGAT-3’ [reverse]). The 2-ΔΔcq method was used to calculate the relative expression level of LINC00922.

### MTT assay and apoptosis analysis

For the MTT assay, cells (1 × 10^3^ cells/well) were seeded in 96-well plates and transfected with shRNAs or controls, and cell proliferation was measured every 1 to 4 days. MTT (20 μl; stock concentration, 5 mg/ml) (Solarbio, China) was added to each well, and the plate was incubated at 37˚C for 4 h. The absorbance at 540nm was measured. For the apoptosis assay, cells were harvested and stained with Annexin V-FITC/PI and analyzed with an Accuri C6 flow cytometer (Becton-Dickinson, USA) and Flow Jo 7.6.1 software (Tree Star Inc., USA).

### Transwell assay and wound-healing assay

The invasion assay was performed using Transwell chambers (Corning, USA). In brief, 1 × 10^5^ cells were added into the upper chamber, and the lower chamber was filled with medium supplemented with FBS (10%, 600 μl), which served as the chemo-attractant, and the plates were incubated for 24h at 37˚C. Next, the cells on the lower membrane were fixed with 100% methanol and stained with 0.1% crystal violet. Five fields were randomly selected and viewed under 200× magnification. The number of cells was counted. For the wound-healing assay, cells (1 × 10^3^) were added into 6-well plates and cultured until confluence. Next, a 200-μL pipette was used to create a wound. The cells were imaged after 24 hours.

### Statistical analysis

GraphPad Prism 6 (USA) was used for statistical analysis. The data were presented as mean ± standard deviation (SD). Student’s t-test was used for comparisons between two groups, and one-way ANOVA was used for comparisons among three or more groups. All experiments were repeated three independent times. P-vales < 0.05 were statistically significant.
